# Wireless Sensor Network-Based Rigid Body Localization for NLOS Parameter Estimation

**DOI:** 10.3390/s22186810

**Published:** 2022-09-08

**Authors:** Pengwu Wan, Jian Wei, Jin Wang, Qiongdan Huang

**Affiliations:** School of Communications and Information Engineering & School of Artificial Intelligence, Xi’an University of Posts and Telecommunications, Xi’an 710121, China

**Keywords:** non-line-of-sight, rigid body localization, time of arrival, concave–convex procedure

## Abstract

In wireless sensor network (WSN)-based rigid body localization (RBL) systems, the non-line-of-sight (NLOS) propagation of the wireless signals leads to severe performance deterioration. This paper focuses on the RBL problem under the NLOS environment based on the time of arrival (TOA) measurement between the sensors fixed on the rigid body and the anchors, where the NLOS parameters are estimated to improve the RBL performance. Without any prior information about the NLOS environment, the highly non-linear and non-convex RBL problem is transformed into a difference of convex (DC) programming, which can be solved by using the concave–convex procedure (CCCP) to determine the position of the rigid body sensors and the NLOS parameters. To avoid error accumulation, the obtained NLOS parameters are utilized to refine the localization performance of the rigid body sensors. Then, the accurate position and the orientation of the rigid body in two-Dimensional space are obtained according to the relative deflection angle method. To reduce the computational complexity, the singular value decomposition (SVD) method is employed to solve the problem in three-Dimensional space. Simulation results show that the proposed method can effectively improve the performance of the rigid body localization based on the wireless sensor network in NLOS environment.

## 1. Introduction

With the rapid development of wireless sensor networks (WSNs), Internet of Things (IoT), and fifth-generation (5G) wireless communication technology, higher precision of the posture information service becomes highly necessary for many types of modern smart devices—for example, unmanned aerial vehicle (UAV) localization, tracking and navigation in the aviation field, posture control of aircraft in the aerospace field, navigation and localization of ships in the maritime field, mechanical arms and robots in industrial production or service equipment, title angle control of instruments in high-precision experiments or medical operations. Obviously, highly precise and robust estimation of the posture parameters, including the position and orientation or the moving direction of the target, is important to guarantee the normal and safe operation of all types of intelligent equipment [1,2].

Different from the traditional “point” source localization scheme [3,4,5], wireless sensor network-based rigid body localization (RBL) technology utilizes an array of sensors mounted on the rigid body, which is conformal with the body. Prior information about the mutual position and the geometric topology of the array is known. The posture information of the rigid body, including the position vector and the orientation matrix, can be determined by using the parameters of wireless signals between the rigid body sensors and the anchors distributed in the surrounding environment.

In recent years, some state-of-the-art results about WSN-based RBL have been presented to precisely estimate the posture parameters of the rigid body. In 2013, Leus constructed the WSN-based RBL model firstly, in which the positions of rigid body sensors in the global coordinate system are taken as a Stiefel manifold, and the least squares (LS) algorithm is employed to determine the position and orientation of the rigid body [1,6]. In reference [7], the maximum likelihood estimation (MLE) problem of RBL is transformed into a semidefinite program (SDP) by using the semidefinite relaxation (SDR) method to determine the posture parameters of the rigid body, which can achieve the Cramer–Rao lower bound (CRLB) at lower noise levels. In addition, the followed research solved the problem of moving rigid body target localization by introducing the Doppler measurement information [8]. Based on the RBL model with distance measurement, reference [2] proposed the divide and conquer (DAC) method and weighted least squares (WLS) method to determine the position of each sensor on the rigid body; then, the initial estimations of the rigid body’s posture parameters are refined to obtain the optimal solution.

Theoretically, due to the correlation between the posture parameters and the position of the sensors, as well as the coupling of the position and orientation of the rigid body, the problem of WSN-based RBL is a non-convex and highly non-linear problem that is difficult to be solved exactly. Therefore, research is increasingly focused on the DAC method to accurately estimate the posture of rigid body, which includes two steps. In the first step, the position of all the rigid body sensors are estimated according to some measurement parameters extracted from the wireless signals transmitted between the sensors and anchors, such as the angle of arrival (AOA) [9], the received signal strength (RSS) [10], the time of arrival (TOA) [11] and the time difference of arrival (TDOA) [12]. Furthermore, some range-free localization approaches also can be employed to obtain the position of the sensors, such as multidimensional scaling map (MDS-MAP) [13], approximate point-in-triangulation Test (APIT) [14], parametric loop division (PLD) [15] and social network analysis (SNA) [16]. Considering the influence of the NLOS error, the range-based algorithm (like TOA) is utilized in this paper, which could provide more accurate localization performance than the range-free method in error environment. In the second step, the position and the orientation parameters of the rigid body are calculated according to the obtained position information of the sensors. This method can avoid the error accumulation in the direct estimation method, but has higher computation complexity.

In order to accurately determine the position information of the rigid body sensors, some mathematical optimization algorithms, such as the modified Newton iteration algorithm (mNIA) [17], participatory searching algorithm (PSA) [18] and particle swarm optimization (PSO) [19], were proposed, respectively, to solve the localization equation. Then, the posture parameters of the rigid body were calculated according to the methods of unit quaternion (UQ) or singular value decomposition (SVD). To obtain better performance, the constrained weighted least squares (CWLS) problem regarding the RBL is relaxed to a convex SDP, which could obtain the global optimal solution of the original problem by using a reasonable second-order cone constraint [20]. The proposed method can achieve accuracy in the CWLS problem at lower noise levels.

The results listed above are obtained in some ideal environment to address the RBL problem, which means that some common error factors are not taken into account, such as the position error of the anchors, the drift of the clock in the time-based method, or even the NLOS error. However, these factors are inevitable in the practical application environment of the WSN-based RBL, which leads to degradation in performance. To overcome the influence of the anchor position error, three different methods were proposed to determine the posture parameters of the rigid body by introducing a calibration emitter [21]. Furthermore, the extended DAC and SDR methods are proposed to solve the RBL problem under the environment of anchor position error without any calibration emitter in the same reference. For the clock error, the SDR method was also employed in the RBL problem based on the TOA measurement in an asynchronous network, which could obtain the clock offset information in the meantime [22].

Unfortunately, the existing results mainly focus on the WSN-based RBL problem in some ideal environment or hardware layout error—for example, the anchor position error or the clock offset. However, the rigid body is mainly applied in urban areas or indoor, with an extremely complex electromagnetic environment, such as robots for massive production and transportation indoors, or unmanned aerial vehicles for emergency rescue in city, where the non-light-of-sight (NLOS) propagation of wireless signals between sensors and anchors is the main factor leading to performance deterioration. Therefore, it is extremely important to study high-precision and robust RBL algorithm in the NLOS environment. We have employed the SDR method to solve the problem in [23], which can overcome the performance deterioration caused by the NLOS error, but needs prior information about the transmitting power and path loss coefficient of the NLOS channel.

Motivated by the abovementioned facts, this paper focuses on the problem of RBL in an NLOS environment by using the cooperative anchor network without any prior information about the transmitter and wireless channel. The main contributions are summarized as follows:

(1) Taking both the sensors’ position and the NLOS error as the parameters to be estimated, the modified concave–convex procedure (CCCP) algorithm is adopted to iteratively refine the estimation performance of the position of the sensors, where the non-convex and highly non-linear rigid body sensor localization equations are converted into the difference of convex (DC) programming based on the model of the WSN-based RBL problem in the NLOS environment.
(2) For different application scenarios, the relative deflection angle method of sensor–anchor pairs and the SVD method are employed to determine the rigid body posture parameters in 2–D and 3–D scenes, respectively. Without any prior information about the NLOS error, computer simulation results show that the proposed method can effectively overcome the effect of the NLOS error upon the estimation of the rigid body’s posture parameters.

The organization of this paper is as follows. Section 2 formulates the RBL problem in NLOS environment. Section 3 proposes the DC programming and the modified CCCP algorithm to determine the positions of sensors and NLOS error parameters. Then, the posture parameters of the rigid body in 2–D and 3–D application scenarios are estimated with different methods. Section 4 simulates and compares the RBL performance of the proposed method with existing methods in the NLOS environment, and Section 5 concludes this paper.

## 2. Problem Formulation

Considering a rigid body in *K*-dimensional (K=2 or 3) space, there are *N* nodes (called sensors in the remainder of the text) are mounted on the body, and the geometric topology is conformal with the rigid body. The position of the sensors in the local coordinate system B is precisely known and denoted as $c_{i}\in R^{K},\;i=1,\;2,\;\ldots ,\;N$. Meanwhile, their positions in the global coordinate system I are unknown and denoted as $s_{i}\in R^{K},\;i=1,\;2,\;\ldots ,\;N$. Theoretically, the relationship between $c_{i}$ and $s_{i}$ can be expressed by using the Stiefel manifold function as [24]

(1) $$s_{i}=Qc_{i}+t,$$

where $Q\in R^{K\times K}$ denotes the rotation matrix of the axis from the local coordinate system B to the global coordinate system I. The parameter $t\in R^{K}$ represents the displacement between the two coordinate systems, namely the translation vector. Both Q and t are the rigid body’s posture parameters to be estimated in the RBL problem. To guarantee the orthogonality of the geometrical coordinate system during the rotation, the rotation matrix Q should satisfy the orthogonality relationship $\mathrm{SO}\left(K\right)=\left\{Q\in R^{K\times K}:Q^{T}Q=I,\det\left(Q\right)=1\right\}$ [25].

In fact, the application of rigid body has different characteristics in different scenarios. For example, for the mechanical arms in industrial production or service equipment, and title angle control of instruments in high-precision experiments or medical operations, they are usually simply and subtly operated in a 2–D plane. The UAVs and aircraft usually fly in 3–D space with complex rotation and displacement. In order to analyze WSN-based RBL in different scenarios of application, localization models are separately constructed in 2–D and 3–D scenarios, which are solved by using different methods in the following section, respectively. In a 2–D scenario, defining the *x*-axis rotation angle of the rigid body around the local coordinate system in the global coordinate system as θ, the rotation matrix Q can be denoted as [21]

(2) $$Q=\left[\begin{array}{cc} \cos\theta & -\sin\theta \\ \sin\theta & \cos\theta \end{array}\right].$$

In a 3–D scenario, the yaw, pitch and roll of the rigid body in the global coordinate system with respect to each axis in the local coordinate system are defined as α, β and γ, respectively. The rotation matrix Q is expressed as

(3) $$Q=\left[\begin{array}{ccc} c_{\beta }c_{\gamma } & -c_{\alpha }s_{\gamma }+s_{\alpha }s_{\beta }c_{\gamma } & s_{\alpha }s_{\gamma }+c_{\alpha }s_{\beta }c_{\gamma }\\ c_{\beta }s_{\gamma } & c_{\alpha }c_{\gamma }+s_{\alpha }s_{\beta }s_{\gamma } & -s_{\alpha }c_{\gamma }+c_{\alpha }s_{\beta }s_{\gamma }\\ -s_{\beta } & s_{\alpha }c_{\beta } & c_{\alpha }c_{\beta } \end{array}\right],$$

where $c_{x}=\cos x$, $s_{x}=\sin x$.

**Figure 1:**
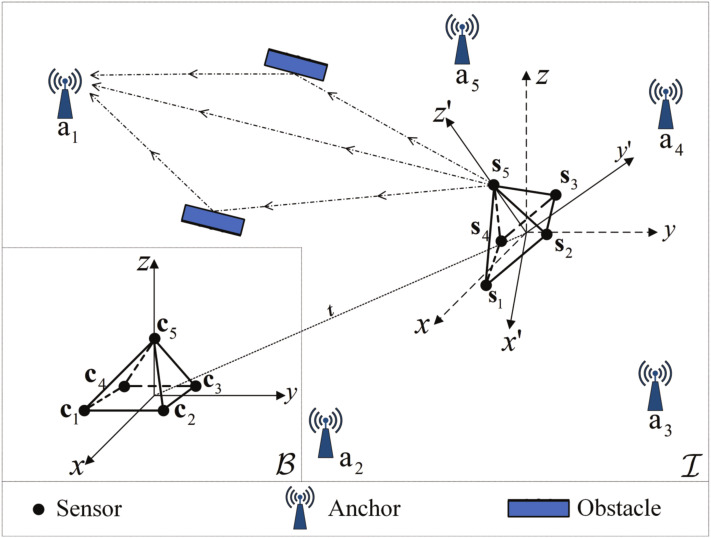
3–D scenario of rigid body localization in the NLOS environment.

In the NLOS environment, rigid body localization in a 3–D scenario is shown in Figure 1. *M* anchors are distributed in the surrounding environment and their precise positions are known at $a_{m}\in R^{K},\;m=1,\;2,\;\ldots ,\;M$ in the global coordinate system. They are employed to precisely determine the rigid body’s posture parameters Q and t. Assuming that both the sensors and anchors have strict clock synchronization, the parameters of TOA measurement regarding the wireless signal in the sensor–anchor pairs can be estimated. The equation for sensor localization can be expressed as

(4) $$d_{mi}=d_{mi}^{o}+n_{mi}+e_{mi}=∥a_{m}-s_{i}∥+n_{mi}+e_{mi},$$

where $d_{mi}$ represents the measurement distance between sensor $s_{i}$ and anchor $a_{m}$, and $d_{mi}^{o}=∥a_{m}-s_{i}∥$ is the real value. $n_{mi}∼N\left(0,\sigma_{mi}^{2}\right)$ is the zero-mean Gaussian measurement noise, assuming that the noise between different sensors is independent of each other. $e_{mi}∼U\left(0,b_{\max}\right)$ is the NLOS error during wireless signal transmission and $b_{\max}$ denotes the upper bound of the NLOS error [26].

By using *M* anchors to jointly determine the rigid body’s posture parameters, the MLE of the RBL problem can be expressed by coupling the above RBL model with the distance measurement Equation (4) as [1]

(5) $$\begin{array}{c} \min_{Q,t}\sum_{m=1}^{M}\sum_{i=1}^{N}\sigma_{mi}^{-2}{\left(d_{mi}-∥a_{m}-Qc_{i}-t∥-e_{mi}\right)}^{2}\\ \;s.t.\;\;Q^{T}Q=I,\det\left(Q\right)=1 \end{array}.$$

As previously mentioned, the position of the sensors in the local and global coordinate systems is used to determine the rigid body’s posture parameters. The rotation matrix Q and translation vector t of the non-regular topology rigid body can be determined by using the topological shape-matching method. However, after rotation of the rigid body with a regular topology such as a square or cube, it is difficult to determine the correspondence among the same sensor in the local and global coordinate systems by the shape-matching method, which results in ambiguity in the orientation parameter estimation. To overcome the ambiguity problem of the sensor matching, one sensor can be taken as the reference node, which transmits the topological information of the sensors to the anchor network. When the anchors determine the position of the reference node in the global coordinate system, the topological information of the sensors transmitted by the wireless signal is extracted to determine the rigid body’s posture parameters. As the problem of orientation ambiguity can be solved by using the hardware method, it will not be considered in the next section.

Due to the strong coupling of the posture parameters of the rigid body and the position of the sensors in the measurement parameters, as well as considering the NLOS error parameter estimation, which make it difficult to be solved, this paper proposes a two-step method to determine the optimal solution of the rigid body’s posture parameters in Section 3. Specifically, to overcome the influence of the NLOS error in the final results, the position of the sensors is estimated in the first step, as well as the NLOS parameters, which can be employed to refine the performance of position of the sensors estimation. In the second step, the  relative deflection angle method among sensor–anchor pairs and the SVD method are introduced to determine the rigid body’s posture parameters in 2–D and 3–D scenarios, respectively.

## 3. Proposed RBL Algorithm

It is obvious from Equation (1) that the posture parameters of the rigid body, including the rotation matrix Q and the translation vector t, are determined by the position of the sensors. In this section, a two-step method is presented to obtain the precise estimation of the RBL, which can avoid the cumulative effect of NLOS error on the RBL performance. In the first step, the objection function of the MLE in (5) is converted into DC programming, which can be solved by using the modified CCCP optimization algorithm to obtain the coordinates of the sensors in the global coordinate system and the NLOS error parameters during the wireless signal transmission. In the processing of the modified CCCP algorithm, the estimated NLOS parameters are iterated to refine the position of the sensors so as to overcome the influence of NLOS errors. In the second step, the relative deflection angle information is utilized to determine the rotation matrix Q and then it is combined with the Stiefel function to solve the translation t in a 2–D scenario. As for the 3–D scenario, the classical SVD method is introduced to determine the posture parameters of the rigid body.

### 3.1. Constructing the DC Programming

To guarantee the integrity of the theoretical process, the definition of the DC programming is given first. Specifically, a continuous function $F\left(x\right)$ is called DC function if it can be expressed as the difference of two convex functions $f\left(x\right)$ and $g\left(x\right)$ in its convex domain D. The programming dealing with the DC function is called DC programming [27]. For variables $\forall x_{1},x_{2}\in D$, one has

(6) $$\begin{array}{c} f\left(x_{2}\right)\leq f\left(x_{1}\right)-{\left(x_{1}-x_{2}\right)}^{T}\nabla f\left(x_{2}\right)\\ g\left(x_{2}\right)\geq g\left(x_{1}\right)+{\left(x_{2}-x_{1}\right)}^{T}\nabla g\left(x_{1}\right) \end{array}.$$

To iteratively minimize the objective function $F\left(x\right)$, assuming that $x^{t}$ and $x^{t+1}$ denote two successive iterative solutions, respectively—for example, $x_{1}=x^{t}$ and $x_{2}=x^{t+1}$. Substituting them into (6), one obtains

(7) $$F\left(x^{t+1}\right)\leq F\left(x^{t}\right)+{\left(x^{t+1}-x^{t}\right)}^{T}\left(\nabla f\left(x^{t+1}\right)-\nabla g\left(x^{t}\right)\right),$$

where $\nabla f\left(x^{t+1}\right)=\nabla g\left(x^{t}\right)$ is the sufficient condition to satisfy the constraint $F\left(x^{t+1}\right)\leq F\left(x^{t}\right)$. Furthermore, taking it as an iterative condition, the CCCP algorithm can find the $x^{t+1}$ to minimize the objective function by using the given $\nabla g\left(x^{t}\right)$.

According to the existing results, if the objective function can be converted into the difference of two convex functions, a non-increasing sequence of iterations can be constructed to find the global optimal solution of the objective function. Therefore, the MLE of the sensor position estimation problem based on TOA measurements in (5) can be expressed as

(8) $$\underset{s_{i}}{\arg\min}\sum_{m=1}^{M}\sum_{i=1}^{N}{\left(d_{mi}-∥a_{m}-s_{i}∥-e_{mi}\right)}^{2}.$$

Furthermore, the objective function in (8) can be expanded and constructed as the difference in $P_{mi}$ and $L_{mi}$, as follows

(9) $$\underset{s_{i}}{\arg\min}\sum_{m=1}^{M}\sum_{i=1}^{N}\underset{P_{mi}}{\underset{︸}{d_{mi}^{2}+{∥a_{m}-s_{i}∥}^{2}+e_{mi}^{2}-2d_{mi}e_{mi}}}-\underset{L_{mi}}{\underset{︸}{2\left(d_{mi}-e_{mi}\right)∥a_{m}-s_{i}∥}}.$$

It is theoretically verified that both $P_{mi}$ and $L_{mi}$ are convex functions, which can be solved directly by using the CCCP algorithm iteratively.

### 3.2. Estimating the Sensor Position

Theoretically, the concave–convex procedure (CCCP) is a monotonic decreasing global optimization method, and its principle is to find out the coordinate point with the closest distance between two convex functions [28]. However, when solving Equation (9) directly by using the CCCP algorithm to determine the position of the sensors, the localization accuracy will inevitably deteriorate due to the NLOS errors.

Therefore, this paper seeks to introduce the idea of iteration into the CCCP algorithm. Firstly, the coordinates of the position of the sensors can be initially determined according to the CCCP algorithm, which contain a large error due to the NLOS error. Secondly, the NLOS error in the wireless signal transmission processing is estimated by using the obtained position of the sensors and the distance measurement information. Thirdly, the estimated NLOS error value is substituted into the original equation to refine the sensor position. Furthermore, to guarantee the performance, an iterative method is employed to ensure the high precision of the sensor position calculation. In the procedure, a precision threshold is set in the iterative algorithm to maintain the computational complexity of the proposed algorithm. The proposed modified CCCP procedure is summarized as follows (Algorithm 1).

It should be pointed out that there is an initial point of the sensor position $s_{i}^{0}$ needed as an input to start the iterative algorithm. In theory, when the initial point is in the convex hull region formed by the anchors, the iterative algorithm can converge globally, which is verified in the simulation section.

**Algorithm 1:** Modified CCCP algorithm
*i*.Initializing the NLOS error $e_{mi}$ and the number of iteration times *t*: $e_{mi}^{0}=0$, t=0;*ii*.Setting the initial iteration point: $s_{i}^{0}$;*iii*.Calculating the gradient factor: $\nabla L_{mi}\left(s_{i}^{t}\right)$;*iv*.Determining the position of the sensors: $s_{i}^{t+1}=\underset{s_{i}}{\arg\min}\sum_{m=1}^{M}P_{mi}\left(s_{i}\right)-s_{i}^{T}\nabla L_{mi}\left({s_{i}}^{t}\right)$;*v*.Determining the NLOS error $e_{mi}^{t+1}$ using the position estimate ${s_{i}}^{t+1}$: $e_{mi}^{t+1}\approx d_{mi}-∥a_{m}-s_{i}^{t+1}∥$;*vi*.Substituting the estimated values of the sensors’ positions $s_{i}^{t+1}$ and the NLOS errors $e_{mi}^{t+1}$ into step iii, setting t=t+1 and repeating the calculation until the accuracy of the positions of sensors satisfies $∥s_{i}^{t+1}-s_{i}^{t}∥<0.1$, outputting the final position estimate $s_{i}$.


### 3.3. Determining the Posture Parameters of the Rigid Body

To reduce the influence of NLOS error, we divided the RBL problem into two steps. In the first step, the NLOS error and the accuracy position of sensors are obtained by using the modified CCCP algorithm, which has been presented in the previous subsection. In the second step, the posture parameters of the rigid body, including the rotation matrix Q and the translation vector t, are calculated according to the obtained position of the sensors in this subsection. According to the characteristics of the rigid body localization problem in 2–D and 3–D scenarios, we propose the relative deflection angle method among sensor–anchor pairs and the SVD method to determine the rigid body posture parameters in 2–D and 3–D scenarios, respectively.

#### 3.3.1. 2–D Scenario

**Figure 2:**
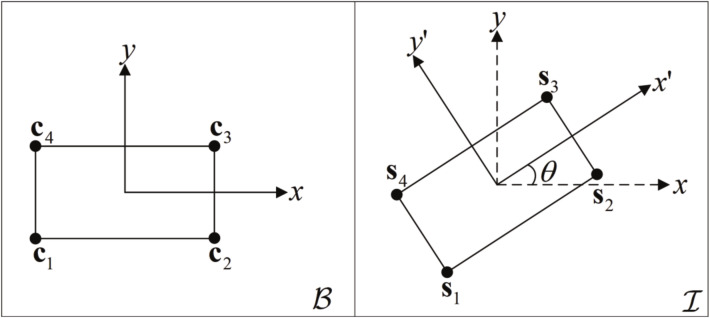
2–D scenario of the rigid body rotation scheme.

In the practical application of RBL in a 2–D scenario, the relationship between the local and global coordinate systems is relatively simple, which means that we can solve the problem with lower computational complexity—for example, in the case of mechanical arms and angle-controlling instruments. The rotation of the rigid body from the local coordinate system B to the global coordinate system I in a 2–D scenario is shown in Figure 2. Obviously, when the position of the sensors is determined, according to the relative deflection angle method, the mathematical geometric relationship between the position of the sensors coordinates and the rotation angle can be employed to solve the rotation matrix Q and translation vector t of the rigid body directly.

With the position of the sensors $c_{i}$ in the local coordinate system and the obtained position $s_{i}$ of the sensors in the global coordinate system, the rotation angle θ of the rigid body can be expressed as

(10) $$\theta =\frac{\sum_{i=1;j=1;i\neq j}^{N}\left[\arctan\left(\frac{s_{y_{i}};-;s_{y_{j}}}{s_{x_{i}};-;s_{x_{j}}}\right)-\arctan\left(\frac{c_{y_{i}};-;c_{y_{j}}}{c_{x_{i}};-;c_{x_{j}}}\right)\right]}{N*\left(N-1\right)}.$$

where $c_{i}={\left[c_{x_{i}},c_{y_{i}}\right]}^{T}$, $c_{j}={\left[c_{x_{j}},c_{y_{j}}\right]}^{T}$ are the coordinate of sensors *i* and *j* in the local coordinate system B, and $s_{i}={\left[s_{x_{i}},s_{y_{i}}\right]}^{T}$, $s_{j}={\left[s_{x_{j}},s_{y_{j}}\right]}^{T}$ are the coordinate of sensors *i* and *j* in the global coordinate system I, respectively.

Substituting the parameter θ into Equation (2), the rotation matrix Q of the rigid body can be obtained. Then, the translation vector t can be calculated according to Equation (1), and one has

(11) $$t=\bar{s}-Q\bar{c},$$

where $\bar{c}$ and $\bar{s}$ are the center of the rigid body in the local and global coordinate systems

(12) $$\bar{c}=\frac{\sum_{i=1}^{N}c_{i}}{N},\;\bar{s}=\frac{\sum_{i=1}^{N}s_{i}}{N}.$$

The proposed method can avoid the influence of quadratic errors in the objective function solution processing and has low complexity. Thus, it can effectively solve the estimation problem of rigid body localization parameters in a 2–D NLOS environment.

#### 3.3.2. 3–D Scenario

In a 3–D localization scenario, such as UAV localization and tracking, navigation and localization of ships in the maritime industry, robots in industrial production or service equipment, the application of RBL is associated with complex rotation and displacement. It is difficult to construct the relative deflection angle equation between the positions of sensors in the local coordinate system B and global coordinate system I, and the method proposed in Section 3.3.1 is unsuitable. Therefore, the singular value decomposition (SVD) method is employed to determine the rigid body’s posture parameters in a 3–D scenario, which is also applicable to a 2–D application scenario.

According to Equation (1), the problem of determining the rotation matrix and translation vector can be formulated as a least squares minimization problem [29]

(13) $$\begin{array}{c} \min_{Q,t}\sum_{i=1}^{N}{∥s_{i}-\left(Qc_{i}+t\right)∥}^{2}\\ \;s.t.\;\;Q\in \mathrm{SO}(K) \end{array}.$$

Setting $\bar{s_{i}}=s_{i}-\bar{s}$, $\bar{c_{i}}=c_{i}-\bar{c}$, and substituting into Equation (13), one has

(14) $$\sum_{i=1}^{N}{∥{\bar{s}}_{i}-Q{\bar{c}}_{i}∥}^{2}=\sum_{i=1}^{N}\left({\bar{s}}_{i}^{T}{\bar{s}}_{i}+{\bar{c}}_{i}^{T}{\bar{c}}_{i}-2{\bar{s}}_{i}^{T}Q{\bar{c}}_{i}\right).$$

Obviously, Equation (14) is minimized when the last term $2{\bar{s}}_{i}^{T}Q{\bar{c}}_{i}$ maximized, which is equivalent to maximizing $\mathrm{Trace}\left(\mathrm{QH}\right)$. The correlation matrix H is defined as

(15) $$H=\sum_{i=1}^{N}{\bar{c}}_{i}{\bar{s}}_{i}^{T}.$$

Assuming that the SVD of matrix $H=U\Lambda V^{T}$, the optimal solution of the rotation matrix is

(16) $$Q=V\cdot \mathrm{diag}\left({\left[1,\det\left(VU^{T}\right)\right]}^{T}\right)U^{T},$$

where diag(**a**) denotes a diagonal matrix formed by the elements of **a**, the length of 1 is K−1 and det(**VU**^T^) ensures that the rotation matrix satisfying det(**Q**) = 1. Since the center of the rigid body also satisfies the relationship in Equation (1), the translation vector **t** can be obtained by using the optimal solution of the rotation matrix, similarly as in (11).

## 4. Complexity Analysis

In this section, we analyze the worst-case computational complexity of the method proposed in this paper for rigid body localization, which is represented by *O*. The complexity of the proposed method in this paper denoted as CCCP-Ref-SVD, comparing with the DAC [2] and SDR [8] methods, as shown in Table 1. In the table, λ represents the number of iterations required to determine the position of the sensors and the NLOS error parameters, and its value range is roughly between 20 and 30, which is obtained in the computer simulation.

As shown in Table 1, since both the DAC method and the SDR method do not consider the effect of NLOS error, the computational complexity is low. During the process of finding the global optimal solution, the proposed method requires alternate iterations to solve the NLOS error parameters and the position of the sensors, resulting in higher computational complexity. However, the proposed method eliminates the effect of NLOS error at the cost of computational complexity and achieves high-precision localization of the rigid body target.

## 5. Simulation Results

In order to fully verify the rigid body localization performance of the proposed method in NLOS environment, the proposed CCCP-Ref algorithm is simulated in 2–D and 3–D scenarios, respectively, with the DAC method [2] and SDR method [8] introduced as the comparison experiments in this section. The simulations are conducted using MATLAB on a personal computer running at 3.3 GHz. The number of anchors is set M=6. With the original global coordinate system I as the center, all anchors are uniformly and randomly distributed in a square (2–D scenario) or a cube (3–D scenario) of length 100 m. The minimum distance between anchors is required to be larger than 20 m.

The Monte Carlo simulation experiments are run L=3000 times, the initial iteration point ${s_{i}}^{0}$ of the sensor position is taken randomly within the convex hull region formed by the anchors, and the NLOS error is uniformly distributed between $\left[0,b_{\max}\right]$. Since the topology of the sensor array is tiny with respect to the anchor array, the NLOS error is assumed to be the same from each anchor to the same sensor, and different for sensors. The parameter settings of each simulation are described before the simulation.

The estimated performance of the NLOS error parameter is denoted as the average deviation (AD), as follows

(17) $$\mathrm{AD}\left(*\right)=\frac{1}{L}\sum_{l=1}^{L}\left|{\left(\hat{*}\right)}^{\left(l\right)}-\left(*\right)\right|,$$

where ${\left(\hat{*}\right)}^{\left(l\right)}$ denotes the estimation of the *l*-th Monte Carlo simulation experiment on $\left(*\right)$, and $\left|\cdot \right|$ represents the absolute value. The localization performance of the rigid body’s posture parameter is expressed by the root mean square error (RMSE), as follows

(18) $$\mathrm{RMSE}\left(*\right)=\sqrt{\frac{1}{L}\sum_{l=1}^{L}{∥{\left(\hat{*}\right)}^{\left(l\right)}-\left(*\right)∥}^{2}},$$

when $\left(*\right)$ is a vector, and $∥\cdot ∥$ represents the Euclidean norm. $∥\cdot ∥$ denotes the Frobenius norm if $\left(*\right)$ is a matrix.

### 5.1. 2–D Scenario

In this subsection, the method proposed in Section 3.3.1 to determine the rigid body’s posture parameters by using the relative deflection angle is simulated and compared with the SVD method in a 2–D scenario, where the localization performance is represented by CCCP-Ref-Deg and CCCP-Ref-SVD, respectively.

Assuming that the local coordinate system B coincides with the global coordinate system I at the initial position, the topological matrix of the sensors in the local coordinate system B is

$$C=\left[\begin{array}{ccccc} 0 & 0 & 2 & 4 & 4\\ -2 & 2 & 0 & -2 & 2 \end{array}\right]m$$

where the parameters in column *i* of C denote the 2–D coordinates of sensor $c_{i}$. In the global coordinate system I, the rigid body is rotated $\theta =30^{\circ }$ around the *x*-axis and the translation vector is set as $t={\left[27,-15\right]}^{T}$ m.

**Figure 3:**
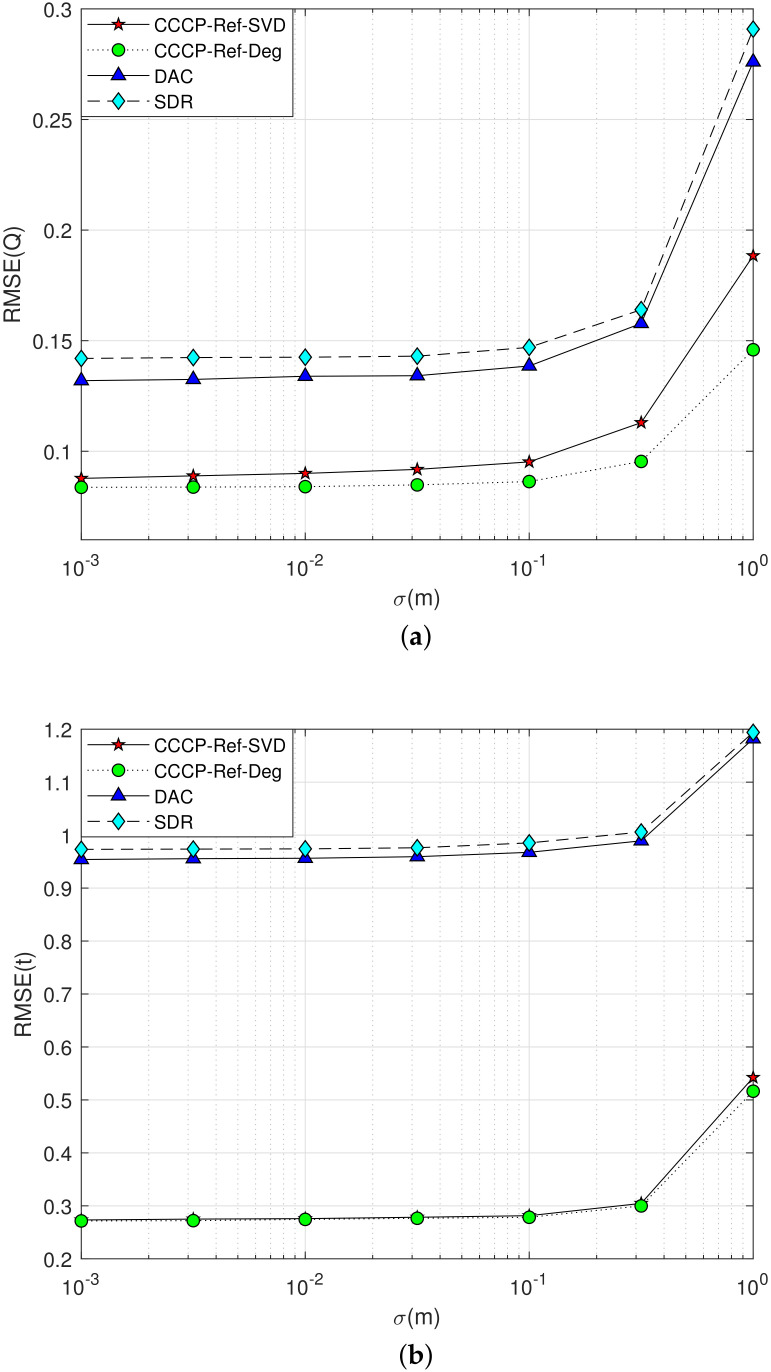
Performance curve of the rigid body’s posture parameter estimation with measurement noise $\sigma_{mi}$ increasing in a 2–D scenario. (**a**) Q Estimation; (**b**) t Estimation.

**Simulation 1**: The maximum value of the NLOS error is set as $b_{\max}=2$ m, the measurement noise $\sigma_{mi}$ varies from $10^{-3}$ m to 1 m, and the performance of rigid body posture parameter estimation is shown in Figure 3.

The estimation performance curves of the rotation matrix Q are shown in Figure 3a, and the translation vector t are shown in Figure 3b. From the simulation results, it can be seen that the localization accuracy of each algorithm decreases with the increasing measurement noise in a 2–D scenario. However, the proposed method has better performance than the DAC method and the SDR method in NLOS environment. That because the NLOS error is taken as the parameter to be estimated and its influence is eliminated in the proposed method. Furthermore, in the case of high measurement noise, the relative deflection angle method proposed in this paper is more accurate than the SVD method to determine the rigid body’s posture parameters in a 2–D scenario. The reason is the increasing error accumulation during the SVD processing.

**Figure 4:**
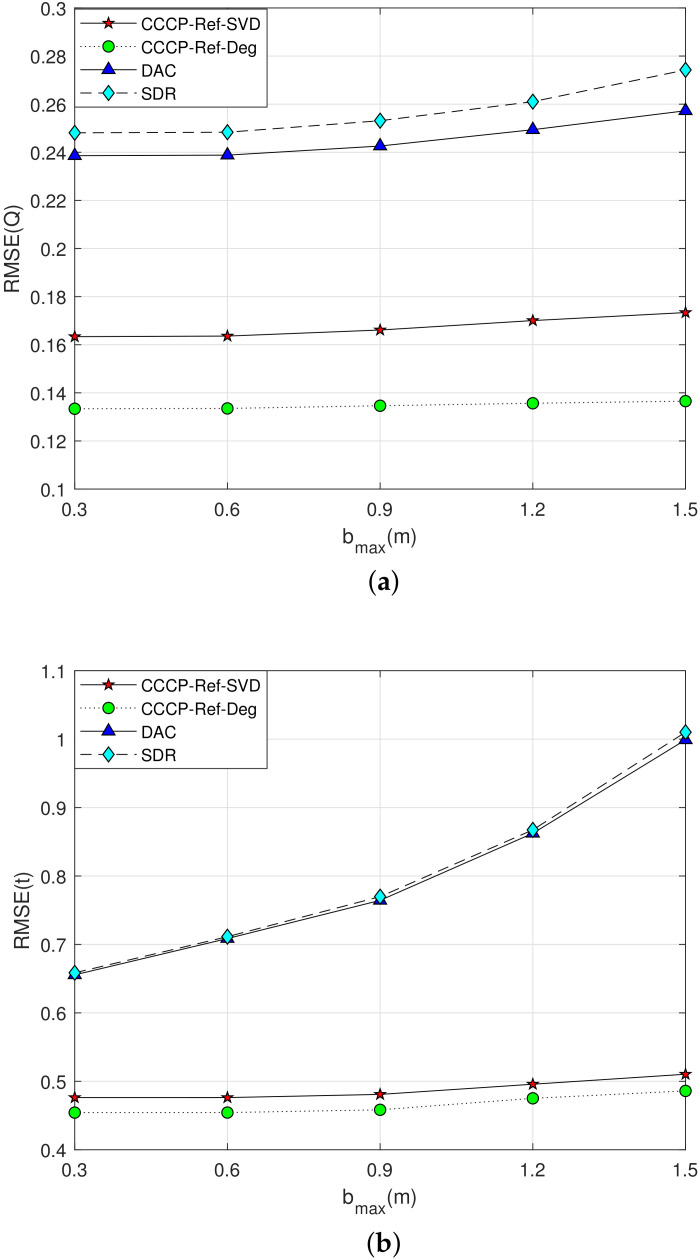
Performance curve of the rigid body’s posture parameter estimation with the maximum value of NLOS error $b_{\max}$ increasing in 2–D case. (**a**) Q Estimation; (**b**) t Estimation.

**Simulation 2**: The measurement noise is set as $\sigma_{mi}=1$ m, the maximum value of NLOS error $b_{\max}$ varies from 0.3 m to 1.5 m, and the estimation performance of the rigid body’s posture parameters is shown in Figure 4.

The estimation performance curves of the rotation matrix Q and the translation vector t are shown in Figure 4a and Figure 4b, respectively. It can be seen that in a 2–D scenario, the localization performance of each compared algorithm decreases severely with the increasing NLOS error. However, the performance of the proposed method tends to be stable in NLOS environment, which demonstrates the robustness of the proposed method for the NLOS error. Similarly, the relative deflection angle method proposed in this paper has better localization performance compared with the SVD method for determining the rigid body’s posture parameters.

### 5.2. 3–D Scenario

Because the relative deflection angle is only applicable to determining the rigid body’s posture parameters in a 2–D scenario, the SVD method is employed in a 3–D localization scenario. Similarly, the local coordinate system B coincides with the global coordinate system I at the initial position, and the topological matrix of the sensors in the local coordinate system B is

$$C=\left[\begin{array}{ccccc} -3 & -3 & 2 & 7 & 7\\ -5 & 5 & 2 & -5 & 5\\ -3 & -3 & 4 & -3 & -3 \end{array}\right]m$$

where the parameters in the column *i* of C represent the 3–D coordinates of sensor $c_{i}$. In the global coordinate system I, the rigid body is rotated by $10^{\circ }$, $25^{\circ }$ and $-20^{\circ }$ around *x*, *y* and *z* coordinate axes, respectively, with the translation vector $t={\left[25,-25,30\right]}^{T}$ m.

**Figure 5:**
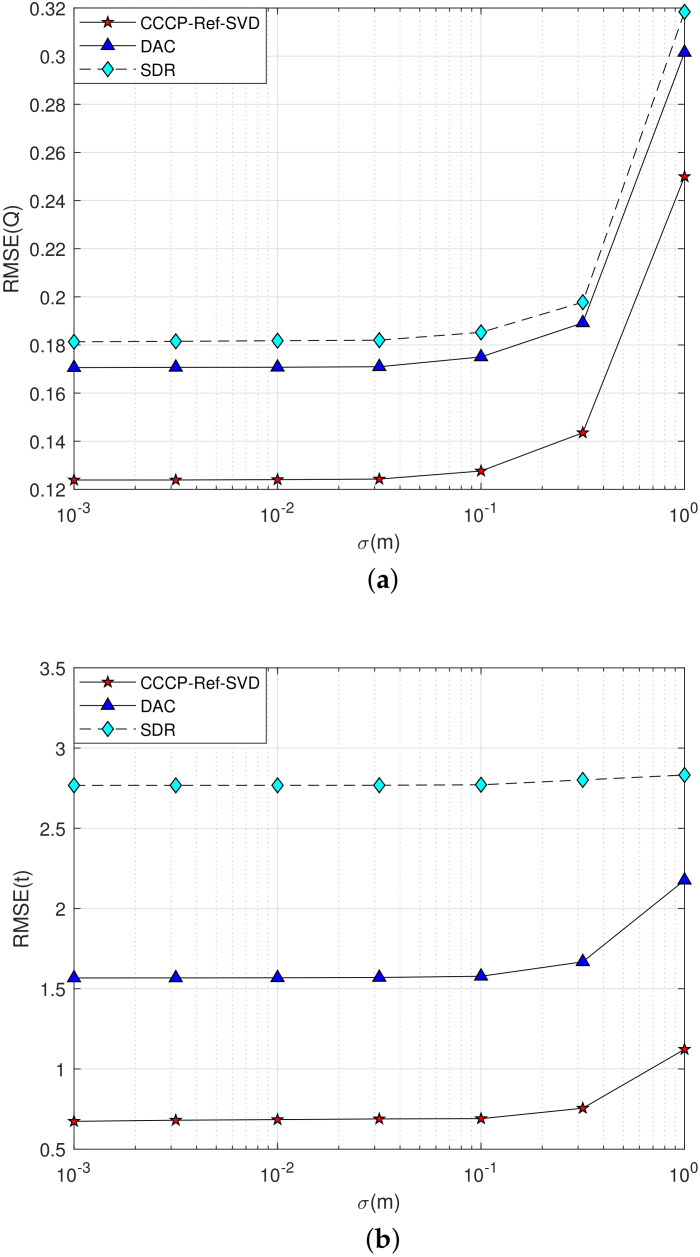
Performance curve of rigid body’s posture parameter estimation with measurement noise $\sigma_{mi}$ increasing in 3–D scenario. (**a**) Q Estimation; (**b**) t Estimation.

**Simulation 3**: The maximum value of the NLOS error is set as $b_{\max}=2$ m, the measurement noise $\sigma_{mi}$ varies from $10^{-3}$ m to 1 m, and the estimation performance of the rigid body’s posture parameters is shown in Figure 5.

As what can be seen from the simulation results in Figure 5a,b, the localization accuracy of each algorithm gradually decreases with the increasing of the measurement noise in the 3–D case. When the measurement noise is larger than $10^{-1}$ m, the estimation performance for each algorithm deteriorates seriously, but the proposed method has better localization performance in NLOS environment compared with the DAC and SDR methods.

**Figure 6:**
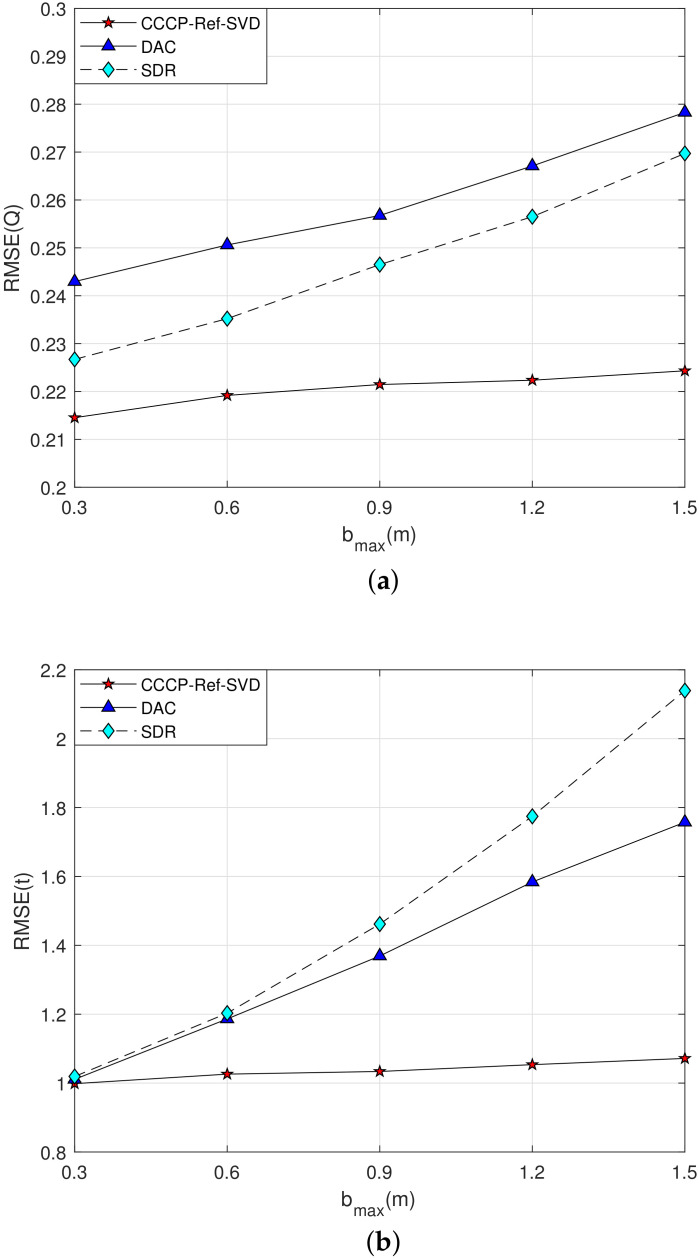
Performance curve of the rigid body’s posture parameter estimation with the maximum value of NLOS error $b_{\max}$ increasing in 3–D scenario. (**a**) Q Estimation; (**b**) t Estimation.

**Simulation 4**: The measurement noise is set as $\sigma_{mi}=1$ m, the maximum value of NLOS error $b_{\max}$ varies from 0.3 m to 1.5 m, and the estimation performance of the rigid body’s posture parameters is shown in Figure 6.

From the simulation results in Figure 6a,b, it can be seen that the localization accuracy of each algorithm decreases gradually with the increasing of the NLOS error in the 3–D case, but the performance of the proposed method in the NLOS environment is superior to the DAC and SDR methods.

Furthermore, the proposed method not only achieves the estimation of the rigid body’s posture parameters in the NLOS environment, but also obtains the value of the NLOS error during the wireless signal transmission. In this simulation, the estimation performance of the NLOS error is verified. The estimated NLOS errors between all anchors and sensors are averaged and expressed as “Estimate”, and “True” denotes the real value. The estimation performance is calculated by using the average deviation indicator. The estimation performance of the NLOS error in simulation 3 and simulation 4 is shown in Table 2 and Table 3, respectively.

The data in the tables show that in the 3–D case, when the maximum value of the NLOS error is constant, the deviation of the NLOS error estimation gradually increases with the increasing of the measurement noise. When the measurement noise is constant, the deviation of the NLOS error estimation tends to be stable with the increasing of the NLOS error maximum value, which demonstrates that the proposed method is mainly affected by the measurement noise and has certain robustness to NLOS error.

## 6. Conclusions

To overcome the influence of the NLOS error on the performance of rigid body localization, the unknown NLOS error is taken as the parameter to be estimated, which is utilized to refine the position of the sensors in the iterative algorithm. Then, the rigid body’s posture parameters are obtained accurately according to different algorithms in a 2–D scenario and 3–D scenario. The computer simulations illustrate that the proposed method can effectively reduce the loss of RBL performance due to the NLOS propagation of wireless signals. In future work, some modern optimization algorithms can be introduced to reduce the computational complexity and provide better performance for the RBL problem in an NLOS environment.

## Figures and Tables

**Table 1:** Comparison of the computational complexity.

Method	Computational Complexity
CCCP-Ref-SVD	$O\left(\lambda MN+K^{3}\right)$
DAC	$O\left(K^{3}+K^{2}MN+MN\right)$
SDR-2-D	$O\left(\sqrt{MN}\left(M^{4}N^{3}+M^{2}N^{2}K^{4}+MNK^{6}\right)\right)$
SDR-3-D	$O\left(\sqrt{MN}\left(M^{4}N^{3}+M^{2}N^{2}K^{4}+2M^{3}N^{2}K^{2}+2MNK^{6}\right)\right)$

**Table 2:** NLOS error estimation results when measurement noise varies in the 3–D case.

σmiσmimm	$10^{-3}$	$10^{-2.5}$	$10^{-2}$	$10^{-1.5}$	$10^{-1}$	$10^{-0.5}$	1
True	1.0037	0.9974	0.9961	1.0046	0.9985	1.0019	1.0024
Estimate	1.0789	1.0732	1.0724	1.0811	1.0752	1.0902	1.1767
AD	0.0752	0.0758	0.0763	0.0765	0.0767	0.0883	0.1743

**Table 3:** NLOS error estimation results when the NLOS error varies in 3–D scenario.

bmaxbmaxmm	0.3	0.6	0.9	1.2	1.5
True	0.1505	0.2990	0.4485	0.6018	0.7509
Estimate	0.3173	0.4678	0.6180	0.7717	0.9269
AD	0.1668	0.1688	0.1695	0.1699	0.1760

## Data Availability

Not applicable.
